# Preparation and Hydrogen Production Application of Core–Shell Heterojunction Photocatalyst (PbS/ZnO)@CuS

**DOI:** 10.3390/ma18010005

**Published:** 2024-12-24

**Authors:** Ming-Huan Chiu, Wein-Duo Yang

**Affiliations:** Department of Chemical and Materials Engineering, National Kaohsiung University of Science and Technology, Kaohsiung 807, Taiwan; titan03@gmail.com

**Keywords:** core–shell structure, (PbS/ZnO)@CuS, photocatalyst, hydrogen production, hydrothermal method

## Abstract

This study employed a hydrothermal method to coat CuS onto PbS quantum dots loaded with ZnO, resulting in a core–shell-structured (PbS/ZnO)@CuS hetero-structured photocatalyst. The sulfide coating enhanced the photocatalyst’s absorption in the near-infrared to visible light range and effectively reduced electron–hole (h^+^) pair recombination during photocatalytic processes. Electron microscopy analysis confirmed the successful synthesis of this core–shell structure using polyvinylpyrrolidone (PVP); however, the spatial hindrance effect of PVP led to a disordered arrangement of the CuS lattice, facilitating electron–hole recombination. Comprehensive analyses using transmission electron microscopy (TEM), photoluminescence (PL), and Brunauer–Emmett–Teller (BET) methods revealed that the (PbS/ZnO)@CuS photocatalyst synthesized at a hydrothermal temperature of 170 °C exhibited optimal hydrogen production efficiency. After conducting a photocatalytic reaction for 5 h in a mixed aqueous solution containing 0.25 M Na_2_S + Na_2_SO_3_ as a sacrificial agent, a hydrogen production rate of 3473 μmol·g^−1^·h^−1^ was achieved.

## 1. Introduction

The rapid development of modern industry and the rapid increase of population have greatly accelerated human consumption of Earth’s resources, especially fossil fuels and forest resources. The remaining useful life is a key factor in maintenance schedules in engine service. Among the energy currently contained in the earth, oil has a remaining useful life of about 47 years, natural gas has a remaining useful life of about 56 years, and coal has a remaining useful life of about 133 years. Moreover, the widespread use of fossil fuels has caused carbon dioxide levels in the atmosphere to reach record highs, intensifying the greenhouse effect, accelerating the melting of polar ice caps, and triggering global climate change. These developments present a significant threat to the sustainable progress of human civilization. Hydrogen generates energy through combustion, does not produce carbon dioxide, and only emits H_2_O. It is recognized as a clean energy source. Currently, countries around the world are actively planning the development of hydrogen energy. Countries such as Japan, Germany, South Korea, and Australia have all announced the development of a hydrogen energy national strategy and have launched a number of large-scale demonstration and verification projects.

According to the report of the International Energy Agency (IEA) [1], if the world is to achieve the vision of net-zero carbon emissions by 2050, the application of hydrogen energy in power generation, vehicles, industry and construction sectors must account for the total global energy. The supply ratio is 13%, so hydrogen energy can be an important energy option to achieve net-zero emissions. According to the IEA “Global Hydrogen Review 2021” report [2], global hydrogen demand in 2020 were approximately 90 million tons, but most of it was produced from fossil fuels, resulting in nearly 900 million tons of carbon emissions. IEA predicts that global hydrogen demand will increase nearly six times to 530 million tons in 2050, of which about 50% will come from heavy industry and transportation sectors such as steelmaking and chemical production; 30% will be used as hydrogen fuel, mainly for shipping, aviation, and injection into existing natural gas pipelines; and 17% will be used for hydrogen-fired power plants to provide stable power with solar photovoltaic and wind power generation.

The Earth receives approximately 1.72 × 10^17^ watts of solar energy per second, of which 1% is enough to supply the needs of the world’s 7.4 billion people for 200 days. If the solar energy received by the Earth’s surface can be effectively utilized and energy efficiency improved, it can serve as a vision to promote a low-carbon environment and ensure the country’s energy sustainability.

Lately, the technology of utilizing semiconductor photocatalysts to transform solar energy into usable energy has become a significant research focus, including the photolysis of water to produce hydrogen [3,4], photocatalyst catalysis [5], degradation of organic pollutants [6], and biomass Energy conversion [7]; these studies contribute to the search for reliable and stable environmental protection and energy solutions. Photocatalyst technology, which harnesses solar energy to split water and produce hydrogen, was initially introduced by Japanese researchers Fujishima Akira and Honda Kenichi in 1972. In this experiment, the researchers used anatase titanium dioxide (Anatase TiO_2_) as the anode electrode and platinum as the cathode electrode to construct a circuit. When exposed to a light source with a wavelength shorter than 415 nm, the titanium dioxide electrode at the anode absorbed light energy. The valence band electrons entered the conduction band under the excitation of light, causing the anode titanium dioxide to react with water to generate oxygen, and at the same time generating hydrogen gas on the cathode platinum electrode. This phenomenon is the famous Honda–Fujishima effect [8].

Photocatalyst technology is regarded as a potential solution to the energy crisis and environmental problems because it can directly use solar energy to decompose water molecules into hydrogen and oxygen. This study reviews the current research status of photocatalytic water splitting for hydrogen production, focusing on the limitations of traditional photocatalyst materials and the recent strategies to use quantum dots and composite semiconductors to improve photocatalytic efficiency. The principle of photocatalyst water decomposition is based on the energy band structure of semiconductor materials. When the photon energy is greater than the energy gap of the semiconductor, electrons will jump from the valence band to the conduction band, and holes will be generated in the valence band. If the conduction band potential is lower than the reduction of water, the potential is more negative, and if the valence band potential is more positive than the oxidation potential of water, then the electrons and holes generate the ability to reduce water to hydrogen and oxidize water to oxygen, respectively.

Although traditional photocatalyst materials, such as TiO_2_, WO_3_, CdS, C_3_N_4_, etc., have good photocatalytic stability, their wide energy gap limits their absorption range of sunlight, and they can only absorb ultraviolet light (<4%); in addition, photoelectrons and holes are easy to combine, reducing photocatalytic efficiency. In order to overcome the above limitations, many studies have proposed a series of improvement strategies, such as noble metal modification [9,10,11]: introducing Pt, Au, Cu and other noble metals or transition metals as cocatalysts, which can effectively promote charge separation and improve yields; and heterogeneous interface [12,13,14]: combining different semiconductor materials to form a heterojunction, which can promote the separation of charges at the interface and improve photocatalytic efficiency, such as CdS@ZnO, CuS/ZnO, ZnO/ZnS, and other materials.

In 1996, Alivisatos et al. published quantum dots (QDs) that controlled and adjusted the size of semiconductor materials through ion exchange [15] to improve the solar light absorption of narrow energy gap semiconductors and improve the bonding process. The problem is to increase the hydrogen production efficiency of photocatalysis. Quantum dots are semiconductor nanocrystals with quantum confinement effect, and their energy gap can be effectively controlled by adjusting their size. Combining quantum dots with traditional photocatalyst materials can broaden the light absorption range and improve the separation efficiency of photogenerated charges, thereby improving photocatalytic performance. The advantages of quantum dots include the tunable energy gaps: by controlling the size of quantum dots, their absorption spectrum can be precisely tuned to match the solar spectrum; the quantum confinement effect: quantum confinement effect enhances light absorption and charge separation; and high surface area: quantum dots have high surface area, providing more active sites.

ZnO is a semiconductor material characterized by a wide band gap, high stability, and low cost, which makes it extensively employed in photocatalytic applications. The photocatalytic performance of ZnO can be significantly enhanced through doping with transition metals or rare earth metals, as well as by constructing heterostructures with other semiconductors. Among its advantages, ZnO exhibits remarkable stability, demonstrating both excellent chemical and thermal stability. Additionally, its relatively low preparation cost contributes to its economic viability. Furthermore, ZnO can be modified through various methods, enabling the optimization of its photocatalytic properties [16,17]. If ZnO is modified to absorb energy in the near-infrared light region, the efficiency of hydrogen production by the photocatalyst can be improved [18].

Compared with single-component nanoparticles, multi-component composite nanoparticles still face many challenges in preparation due to their structural complexity, and there are relatively few related studies [19]. However, the unique properties and diverse application potential exhibited by multi-component composite particles have made it a research hotspot in the field of nanomaterials. Multi-component composite nanoparticles can be divided into mixed composite particles: different components are evenly dispersed inside the entire particle, like a piece of floral cloth, with threads of various colors intertwined; core–shell composite particles: like an onion, from the inside out, have a multi-layer structure on the outside, and the composition of each layer is different [20]. In recent years, core–shell composite particles have received widespread attention due to their precisely controllable structure and properties. Scientists have successfully prepared various types of core–shell composite nanoparticles, such as core–shell-structured quantum dots (ex: CdSe/ZnS) and nano metal shells (ex: SiO₂/Au).

Nandi et al. incorporated ZnO and CdS nanoparticles (NPs) onto a CuS photocatalyst and modified the energy band alignment of the photocatalyst through the heterostructure, significantly enhancing the efficiency of photocatalytic degradation [21]. Shi et al. first synthesized PbS to create QDs, which were then used to prepare a PbS@ZnO/graphene oxide photocatalyst. The multiple excitation effects of PbS QDs generate a strong synergy with graphene oxide, effectively aiding in the separation of electron–hole pairs and enhancing the efficiency of photocatalytic hydrogen production [22]. Liu et al. prepared (ZnS-PbS)/Au/TiO_2_ photocatalyst by hydrothermal method. The loaded PbS QDs can increase the absorption range of the photocatalyst from the near-infrared light region to the visible light region, using 20 wt.% methanol as the sacrificial reagent. Using hydrogen production, the efficiency reached 5011 μmol·g^−1^·h^−1^ [23].

Wang et al. electrochemically modified the ZnO rod/reduced graphene rGO/CdS composite material with CuS at room temperature to obtain a good heterostructure material. Studies have shown that the addition of CuS nanoparticles plays a key role in enhancing visible light response and exhibits excellent catalytic performance; visible light was obtained in the CuS-ZnO/rGO/CdS heterostructure containing 0.23 at% CuS and 1.62 at% CdS. The photocatalytic H_2_ generation rate was 1073 μmol·g^−1^·h^−1^ [24].

From the abovementioned research results, it is known that supporting narrow bandgap semiconductors on the photocatalyst substrate through heterogeneous bonding can improve the photocatalytic efficiency of the photocatalyst. Therefore, this study loaded a CuS shell and PbS QDs on the surface of ZnO photocatalyst and produced a core–shell morphology. The energy gap relationship of the photocatalyst was improved by loading sulfide with a narrow energy gap, and the photocatalyst’s performance in the visible and near-infrared regions was increased. The absorption range was increased to improve the photocatalytic hydrogen production efficiency of the photocatalyst.

## 2. Experimental Materials and Experimental Methods

### 2.1. Experimental Reagent

The chemicals used in this study, along with their purities, were as follows: zinc acetate (Showa, 99%, Tokyo, Japan), copper nitrate (ACROS, 99%, Geel, Anvers, Belgium), lead acetate (Alfa Aesar, 99%, Heysham, UK), thioacetamide (Alfa Aesar, 99%, Essex, MA, USA), polyvinylpyrrolidone (PVP, VETEC, 99%, Speyer, Rheinland-Pfalz, Germany), sodium sulfide (Acros, 98+%, Geel, Anvers, Belgium), sodium sulfite (Showa, 97%, Tokyo, Japan), sodium hydroxide (Showa, 97%, Tokyo, Japan), and ammonia (Showa, 28%, Tokyo, Japan).

#### 2.1.1. FE-STEM Analysis

The photocatalyst was subjected to ultrasonic vibration in a methanol solution for 1 h, then the solution was drop-cast onto a 400-mesh carbon-coated copper grid. The grid was then dried in an oven at 60 °C for approximately 24 h. After the sample was completely dry and solvent-free, it was subjected to vacuum drying for 30 min. Following this, FE-STEM analysis was conducted.

#### 2.1.2. XRD Analysis

The analysis was conducted using a copper target (CuKα, λ = 1.5406 Å) with an output voltage of 40 kV and an output current of 40 mA. The measurement range was from 20° to 80°, with a scanning rate of 4°/min.

#### 2.1.3. UV-Vis-NIR Analysis

The photocatalyst powder was evenly spread and placed onto a substrate containing quartz glass, with an integrating sphere used for measurement. The scanning range was from 300 to 1900 nm.

#### 2.1.4. Photoluminescence (PL) Analysis Conditions

The photocatalyst was illuminated with a light source of 610 nm wavelength, and the fluorescence intensity resulting from the recombination of electron–hole pairs, after the electrons were excited to the conduction band and then relaxed to the valence band, was detected. This allows the assessment of the recombination degree of the electron–hole pairs in the photocatalyst. The total measurement range was from 330 to 620 nm.

### 2.2. Characterization of Photocatalysts

Prior to photocatalytic analysis, the sample was first placed in a freeze-dryer at −50 °C for 24 h, followed by drying in an oven at 105 °C for 24 h to eliminate any residual moisture and low-boiling components. The surface structure and particle morphology of the photocatalyst were examined using Field Emission Scanning Transmission Electron Microscopy (FE-STEM) (Talos, F200X G2, Prague, Czech Republic). The crystal phase of the material was identified through X-Ray Powder Diffraction (XRD; BRUKER, D8 Discover, Bremen, Germany). The optical properties of the photocatalyst are investigated via Ultraviolet-Visible-Near-Infrared Diffuse Reflectance Spectroscopy (UV-Vis-NIR) (JASCO; V-670, Nagoya City, Japan). Fluorescence spectroscopy (Hitachi, F-4500, Chiyoda City, Japan) was used to assess the fluorescence intensity, which helped determine the recombination rate of electron–hole pairs. Finally, the hydrogen production rate of the photocatalyst was measured using a Gas Chromatograph (GC, 6500GC system, YL Instrument Co., LTD., Anyang, Korea) equipped with a Thermal Conductivity Detector (TCD).

### 2.3. Preparation of ZnO Nanomaterials

The ZnO used in this study was synthesized via the precipitation method. Initially, 3 g of Zn(CH₃COO)₂∙2H₂O was dissolved in 100 mL of deionized (DI) water. The resulting solution was heated to 80 °C and continuously stirred at 300 rpm using a magnetic stirrer for 30 min. At the same time, 20 mL of dilute NH₃ solution was added dropwise at a rate of 1–2 mL per minute, and stirring was continued for an additional 2 h. Simultaneously, 20 mL of dilute NH₃ solution was added drop by drop at a rate of 1–2 mL per minute, and stirring was maintained for another 2 h. The precipitate was subsequently washed several times with DI water and ethanol (C₂H₅OH) before being freeze-dried for 8 h. After the drying process was finished, the ZnO photocatalyst powder was placed in a sample bottle and sealed with aluminum foil to shield it from external light exposure.

### 2.4. PbS QDs Loaded onto ZnO Photocatalyst

A suitable amount of Pb(CH₃COO)₂∙3H₂O was weighed and dissolved in deionized water to form an acetate solution. Subsequently, the ZnO photocatalyst solution, which had been pre-dispersed in deionized water, was added to the acetate solution, and the mixture was stirred and shaken thoroughly to ensure uniform mixing. An excess of CH₃CSNH₂ (2.5 times the mole amount of lead acetate) was then added at a rate of 1–2 mL min^−1^, followed by stirring for 2 h and ultrasonic agitation for 30 min. The resulting mixture was transferred to a Teflon-lined autoclave and heated at a rate of 10 °C min^−1^ to 130 °C for hydrothermal treatment lasting 16 h. After completion of the reaction, the obtained powder was washed with deionized water and ethanol, followed by freeze-drying, resulting in the formation of a PbS/ZnO photocatalyst.

### 2.5. Preparation of (PbS/ZnO)@CuS Photocatalyst via Hydrothermal Method

**Figure 1:**
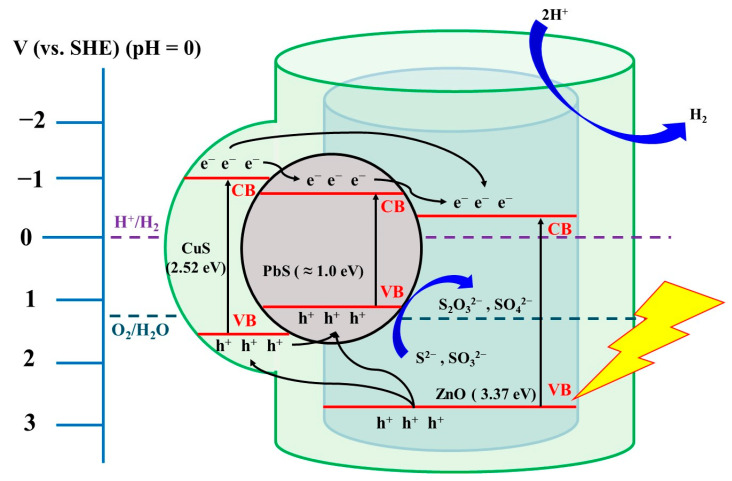
Bandgap relationship of the synthesized core–shell-structured (PbS/ZnO)@CuS heterojunction photocatalyst.

A suitable amount of Cu(NO₃)₂·3H₂O was weighed and dissolved in deionized water to form a nitrate solution. This solution was then added to the PbS/ZnO photocatalyst solution, which had been pre-dispersed in deionized water containing polyvinylpyrrolidone (PVP). The mixture was stirred and shaken thoroughly to ensure uniform mixing. Subsequently, an excess of CH₃CSNH₂ (2.5 times the mole amount of copper nitrate) was added at a rate of 1–2 mL min^−1^, and the pH was adjusted to 11 using 1 M NaOH. The mixture was stirred for 2 h and subjected to ultrasonic agitation for 30 min to facilitate the reaction. The resulting mixture was transferred to a Teflon-lined autoclave and heated at a rate of 10 °C min^−1^ to 130/150/170/190 °C, undergoing hydrothermal treatment for 24 h. After completion of the reaction, the obtained powder was washed with deionized water and ethanol, followed by freeze-drying, resulting in the formation of the (PbS/ZnO)@CuS photocatalyst. Figure 1 illustrates the bandgap relationship of the prepared core–shell-structured (PbS/ZnO)@CuS heterojunction photocatalyst.

### 2.6. Synthesis of (PbS/ZnO)@CuS Photocatalyst by Hydrothermal Method

We mixed the 0.02 M PbS/ZnO photocatalyst aqueous solution with the polyvinylpyrrolidone (PVP) aqueous solution prepared with deionized water and then stirred and mixed with the 0.02 M Cu(NO₃)₂·3H₂O magnet for 15 min. The mixture was stirred and shaken thoroughly to ensure uniform mixing. Subsequently, an excess of CH₃CSNH₂ (2.5 times the mole amount of copper nitrate) was added at a rate of 1–2 mL min^−1^, and the pH was adjusted to 11 using 1 M NaOH. The mixture was stirred for 2 h and subjected to ultrasonic agitation for 30 min to facilitate the reaction. The resulting mixture was transferred to a Teflon-lined autoclave and heated at a rate of 10 °C min^−1^ to 130/150/170/190 °C, undergoing hydrothermal treatment for 24 h. After completion of the reaction, the obtained powder was washed with deionized water and ethanol, followed by freeze-drying at −50 °C and keep 24 h to remove water from the samples, resulting in the formation of the (PbS/ZnO)@CuS photocatalyst. The molarity ratio of ZnO@PbS:CuS is 1:1. Figure 1 illustrates the bandgap relationship of the prepared core–shell-structured (PbS/ZnO)@CuS heterojunction photocatalyst.

### 2.7. The Degree of Recombination of Photocatalyst

The degree of recombination of the photocatalyst refers to the electron–hole recombination rate of the synthesized photocatalyst, measured using photoluminescence (PL) via fluorescence spectroscopy. When the photocatalyst is exposed to light with a wavelength of 579 nm, the excited photocatalyst generates electrons in the conduction band, which then relax back to the valence band, leading to the emission of fluorescence. The recombination rate of the electron–hole pairs in the photocatalyst was assessed by analyzing the fluorescence intensity of each sample.

### 2.8. Photocatalytic Hydrogen Production

The photocatalytic water-splitting hydrogen production reactor system employed in this study was designed as a semi-open type. A 300 W solar simulator served as the simulated light source, equipped with an AM 1.5G filter (SF-300-A, SCIENCETECH, London, Ontario, Canada) to provide appropriate illumination. The distance between the light source and the photocatalytic reactor was maintained at 20 cm. The reactor’s main body was constructed from light-transmitting quartz glass, featuring four openings: two for the inlet and outlet of helium gas, one for pressure detection, and one for sampling.

Fifty milligrams of (PbS/ZnO)@CuS photocatalyst powder was placed in the reactor, and the photocatalytic reaction was carried out in pure water to examine the effects of hydrogen production through water splitting. This was compared to results obtained with the addition of a 0.25 M sacrificial reagent, composed of a mixed solution of Na₂S and Na₂SO₃. After the addition of the 0.25 M sacrificial reagent, the photocatalytic reaction was allowed to proceed for 5 h, followed by centrifugation at 6000 rpm to separate the photocatalyst from the solution. The water-splitting experiment was repeated 10 times under the same conditions.

During the photocatalytic water splitting for hydrogen production, gas samples were collected from the reactor every hour using a gas syringe and analyzed for hydrogen content with a gas chromatograph (GC, YL Instrument 6500GC system) equipped with a thermal conductivity detector (TCD). Helium was used as the carrier gas at a flow rate of 30 mL per minute. The samples were injected into the injection port at a temperature of 180 °C, and the molecular sieve column was kept at 60 °C.

## 3. Results

### 3.1. Analysis of Material Properties

#### 3.1.1. STEM

**Figure 2:**
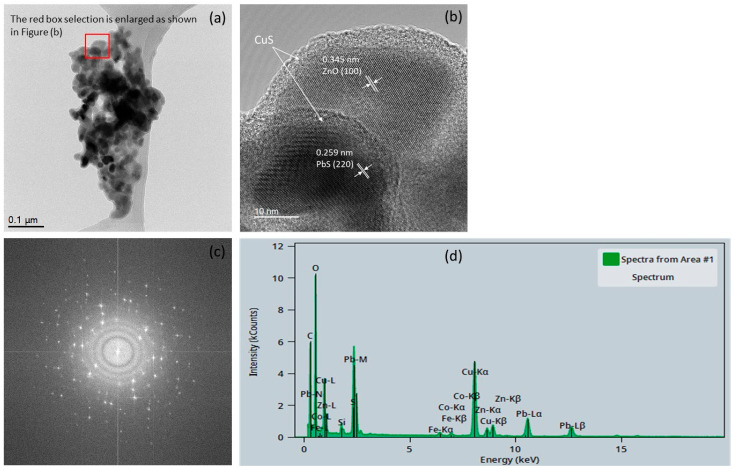
TEM analysis of the (PbS/ZnO)@CuS heterojunction photocatalyst: (**a**) image of the (PbS/ZnO)@CuS photocatalyst, (**b**) HR-TEM image of the (PbS/ZnO)@CuS photocatalyst at a higher magnification, (**c**) electron diffraction, (**d**) EDX analysis.

The scanning transmission electron microscope (STEM) was utilized to analyze the heterojunction photocatalyst, as illustrated in Figure 2. From Figure 2a,b, it is evident that the synthesized photocatalyst features a complete coating of CuS over the PbS/ZnO core, confirming the successful preparation of the core–shell-structured (PbS/ZnO)@CuS photocatalyst. The core, consisting of PbS/ZnO, was synthesized following the previously established hydrothermal method, and the PbS quantum dots exhibited a nearly spherical morphology [23,25].

Figure 2b presents a magnified view of Figure 2a, clearly revealing a lattice spacing of 0.345 nm for ZnO, corresponding to the (100) crystal plane of hexagonal wurtzite. In contrast, the QDs-PbS displays a lattice spacing of 0.259 nm, indicative of the (220) crystal plane [5,26]. However, CuS does not exhibit a distinct lattice arrangement, which may be attributed to the synthesis process. To fabricate the shell structure of CuS, PVP was employed as a dispersing agent during the preparation of the (PbS/ZnO)@CuS photocatalyst. Consequently, the spatial hindrance effect induced by PVP likely led to a disordered lattice arrangement when S^2−^ ions combined with Cu^2^⁺ ions to form CuS [27,28].

Additionally, electron diffraction analysis (Figure 2c) revealed that the (PbS/ZnO)@CuS photocatalyst material has a polycrystalline composition. The observation of a halo effect in the photocatalyst material suggests that the nucleation of CuS is influenced by the spatial hindrance effect of PVP, resulting in a disordered atomic arrangement [27,28]. Energy dispersive X-Ray spectroscopy (EDX) analysis, shown in Figure 2d, clearly indicates the presence of Zn, Pb, Cu, O, and S elements in the spectrum, confirming the successful synthesis of the (PbS/ZnO)@CuS photocatalyst.

#### 3.1.2. Element Distribution Analysis

**Figure 3:**
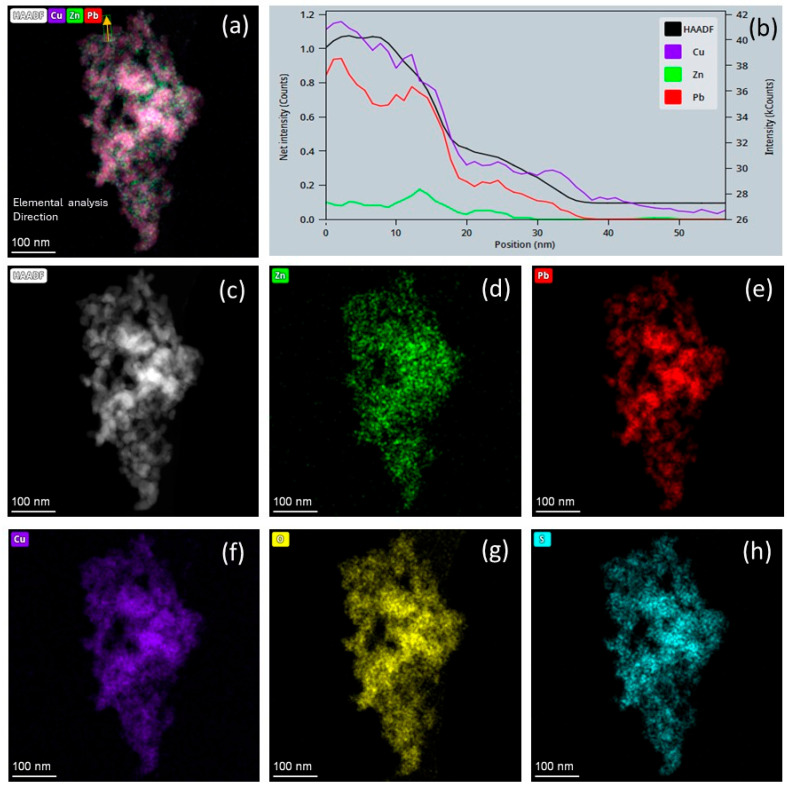
(PbS/ZnO)@CuS heterojunction photocatalyst: (**a**) distribution of all elements, (**b**) profile, (**c**) HAADF, (**d**) Zn, (**e**) Pb, (**f**) Cu, (**g**) O, (**h**) S.

Figure 3b represents the intensity of the elemental signals indicated by the arrows in Figure 3a. It can be observed that, due to the core–shell structure of the (PbS/ZnO)@CuS photocatalyst, the signal intensity of the elements is highest for Cu, followed by Pb, with Zn being the lowest. Figure 3d–h show the distribution maps of each element, clearly displaying the presence of Zn, Pb, Cu, O, and S elements on the (PbS/ZnO)@CuS photocatalyst.

#### 3.1.3. SEM

**Figure 4:**
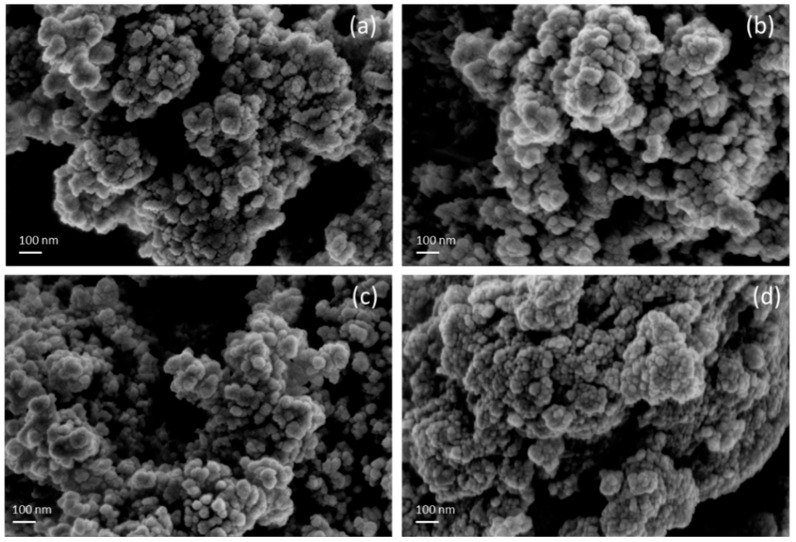
SEM images of (PbS/ZnO)@CuS photocatalysts at different hydrothermal temperatures: (**a**) 130 °C, (**b**) 150 °C, (**c**) 170 °C, (**d**) 190 °C.

Figure 4 presents SEM images of the (PbS/ZnO)@CuS photocatalysts prepared at different hydrothermal temperatures. As the hydrothermal temperature increases, the agglomeration of the photocatalysts becomes more pronounced [29]. The appearance of the photocatalysts at all temperatures is characterized by interconnected, approximately spherical shapes, with no significant differences observed. In contrast to the CuS/PbS/ZnO photocatalysts synthesized by Chiu et al. [25], no distinct hexagonal wurtzite ZnO (rectangular) shapes were observed in Figure 4a–d. This is attributed to the requirement of a higher concentration of CuS for coating the PbS/ZnO photocatalysts to achieve the core–shell structure, resulting in photocatalysts that appear as interconnected spheres, which is consistent with previous TEM analysis results.

#### 3.1.4. XRD

**Figure 5:**
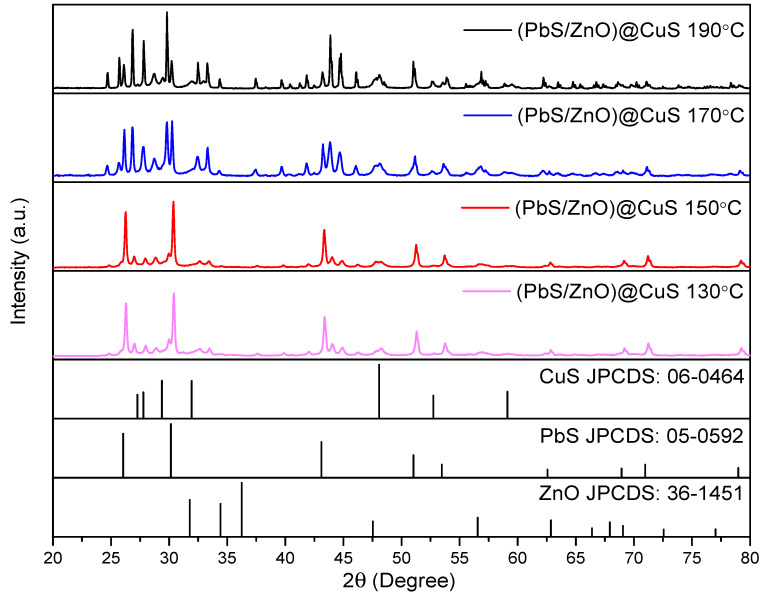
XRD analysis of (PbS/ZnO)@CuS photocatalysts synthesized at different hydrothermal temperatures.

The X-ray diffraction (XRD) patterns of the (PbS/ZnO)@CuS photocatalysts synthesized at various hydrothermal temperatures are presented in Figure 5. Characteristic peaks of hexagonal wurtzite ZnO are observed at 2θ values of 31.8°, 34.5°, 36.3°, 47.6°, 56.6°, 62.9°, 66.4°, 68.0°, and 69.1° [30]. Additionally, the PbS quantum dots exhibit characteristic peaks at 2θ values of 26.0°, 30.1°, 43.1°, 51.0°, 53.5°, 62.6°, 68.9°, 70.9°, and 78.9°, corresponding to the (111), (200), (220), (311), (222), (400), (331), (420), and (422) crystal planes, respectively, which align with the JCPDS card number 05-0592 [5]. The shell layer, CuS, presents distinct peaks at 2θ values of 27.3°, 27.8°, 29.4°, 48.1°, and 59.1°, corresponding to the (100), (102), (103), (108), and (116) crystal planes, consistent with JCPDS card number 06-0464 [31]. The analysis confirms the successful synthesis of the (PbS/ZnO)@CuS photocatalyst, as evidenced by the characteristic peaks of each material. Notably, the peaks of CuS at 48.1° overlap with the ZnO peak at 47.6°, resulting in an enhanced signal. As the hydrothermal temperature increases, the intensity of the characteristic peaks of the (PbS/ZnO)@CuS photocatalyst also increases, indicating a more complete formation of the sulfide phases [32].

### 3.2. Spectral Properties of Prepared Heterostructure Photocatalysts

#### 3.2.1. UV-Vis-NIR Absorption Characteristics

**Figure 6:**
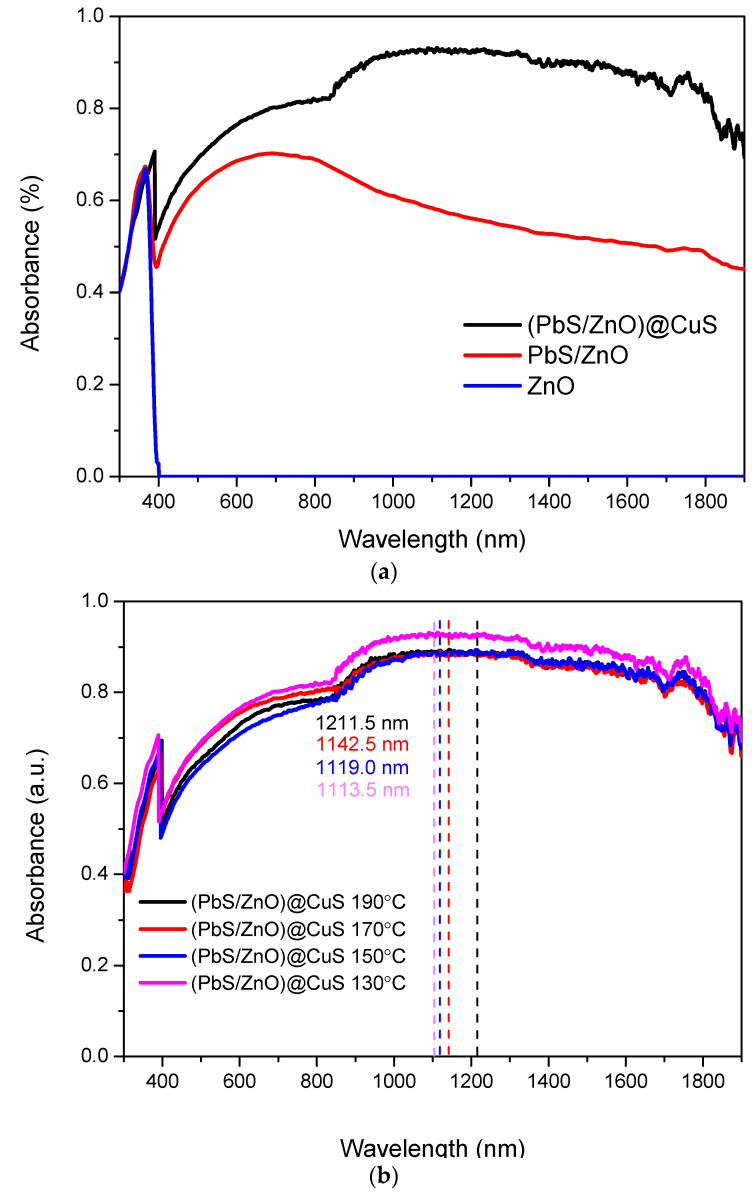
Absorption spectra of the photocatalysts: (**a**) ZnO, PbS, (PbS/ZnO)@CuS; (**b**) (PbS/ZnO)@CuS at hydrothermal temperatures (130–190 °C).

The UV-Vis-NIR absorption characteristics of ZnO, PbS/ZnO, and (PbS/ZnO)@CuS photocatalysts, as shown in Figure 6a, illustrate the UV-Vis-NIR absorption spectra of ZnO, PbS/ZnO, and (PbS/ZnO)@CuS photocatalysts. The unmodified ZnO exhibits an absorption peak beginning at approximately 400 nm [33]. Following the loading of PbS quantum dots onto ZnO, the PbS/ZnO photocatalyst demonstrates significant absorption across the near-infrared to visible light range [34]. The synthesized (PbS/ZnO)@CuS photocatalyst, featuring a core–shell structure, further enhances the absorption performance in this spectral region [5,25].

Additionally, Figure 6b compares the UV-Vis-NIR absorption characteristics of (PbS/ZnO)@CuS photocatalysts prepared at different hydrothermal temperatures. The positions of the maximum absorption wavelengths (dashed lines) for the sulfides synthesized at various hydrothermal temperatures indicate that lower hydrothermal temperatures result in smaller quantum dot sizes, leading to a blue shift in the absorption wavelengths [23,25,35]. Specifically, the maximum absorption wavelength shifts from 1211.5 nm at a hydrothermal temperature of 190 °C to 1113.5 nm at 130 °C. This indicates that under lower hydrothermal temperature conditions, the prepared photocatalysts possess smaller quantum sizes, which widen the bandgap due to the quantum size effect, thereby resulting in a blue shift in the absorption spectrum. This finding is consistent with the results of previous studies by Liu et al. and Chiu et al. [23,25].

#### 3.2.2. Tauc Curve Analysis

**Figure 7:**
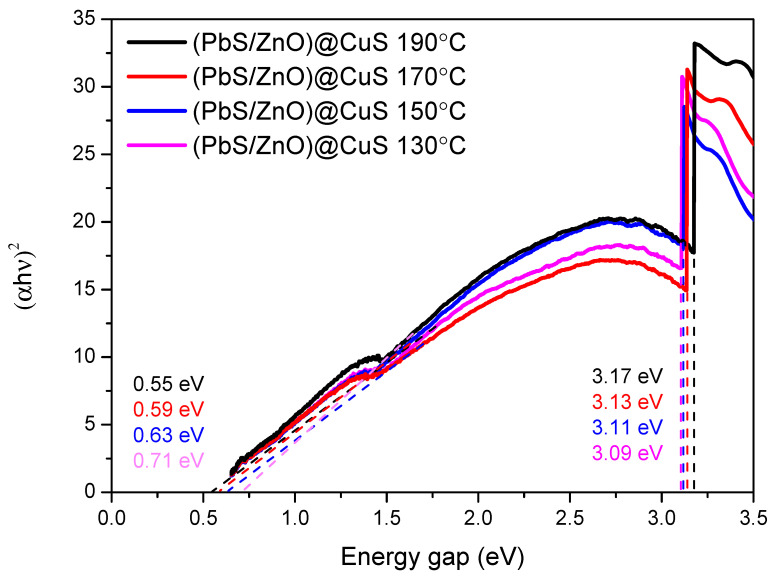
Tauc analysis of (PbS/ZnO)@CuS photocatalyst at different hydrothermal temperatures.

Through Tauc curve analysis, this study investigates the band gap relationship of the synthesized (PbS/ZnO)@CuS photocatalyst at different hydrothermal temperatures (as shown in Figure 7). The photocatalyst exhibits two band gaps, with the lower band gap corresponding to PbS and the higher band gap corresponding to ZnO. It was observed that as the hydrothermal temperature increased from 130 °C to 190 °C, the ZnO band gap of the (PbS/ZnO)@CuS photocatalyst increased from 3.11 eV to 3.18 eV, while the band gap of PbS quantum dots decreased from 0.71 eV to 0.55 eV. The band gap of the PbS quantum dots synthesized at a hydrothermal temperature of 130 °C was approximately 0.71 eV, which is consistent with the results obtained by Chiu et al. in their hydrothermal synthesis of PbS/ZnS photocatalysts, indicating that the size of the PbS quantum dots is correlated [25,36]. Additionally, Guchhait et al. noted that smaller PbS quantum dot particles possess larger band gaps, which enhances the photocatalyst’s absorption from the near-infrared region into the visible light region, resulting in a blue shift phenomenon [37].

#### 3.2.3. Photoluminescence (PL) Analysis Results

**Figure 8:**
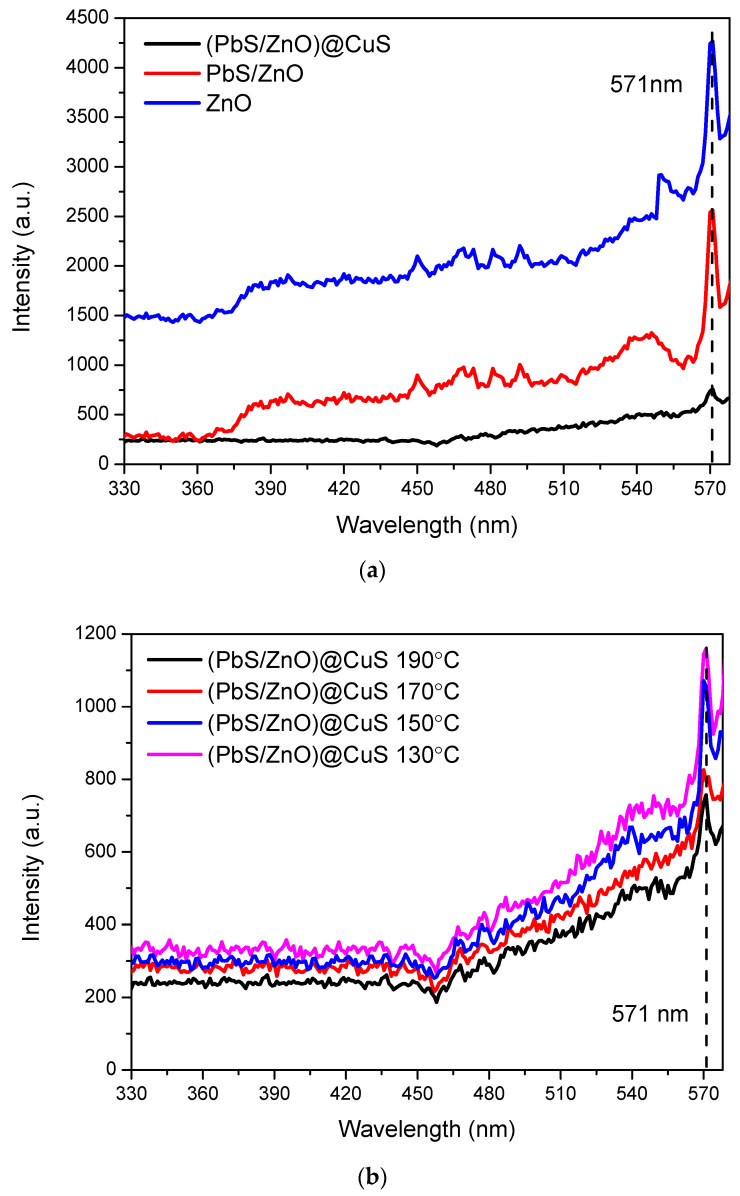
PL spectra of the photocatalysts: (**a**) ZnO, PbS, (PbS/ZnO)@CuS; (**b**) (PbS/ZnO)@CuS at hydrothermal temperatures (130–190 °C).

Through photoluminescence (PL) analysis, this study investigated the recombination degree of electron–hole pairs in the prepared photocatalysts. As shown in Figure 8a, all synthesized photocatalysts exhibited a peak around 571 nm, with unmodified ZnO displaying the highest fluorescence intensity. In contrast, the (PbS/ZnO)@CuS photocatalyst, obtained by loading ZnO with PbS quantum dots and subsequently covering it with CuS, demonstrated the lowest fluorescence intensity. This indicates that the surface treatment of the ZnO photocatalyst with sulfides altered the interfacial bonding of the heterojunction, effectively separating the electrons and holes and reducing the occurrence of recombination [38].

The fluorescence intensity of the (PbS/ZnO)@CuS photocatalyst prepared at different hydrothermal temperatures is shown in Figure 8b. It was observed that the fluorescence intensity of the photocatalyst gradually decreased with increasing hydrothermal temperature. Specifically, the photocatalyst synthesized at 190 °C exhibited the lowest fluorescence intensity, while the highest intensity was observed at 130 °C. This phenomenon is attributed to the lower crystallinity and the presence of lattice defects in the sulfides produced at lower hydrothermal temperatures, which makes recombination more likely when the photocatalyst is excited to generate electron–hole pairs [25,39].

### 3.3. Analysis of Hydrogen Production Efficiency

**Figure 9:**
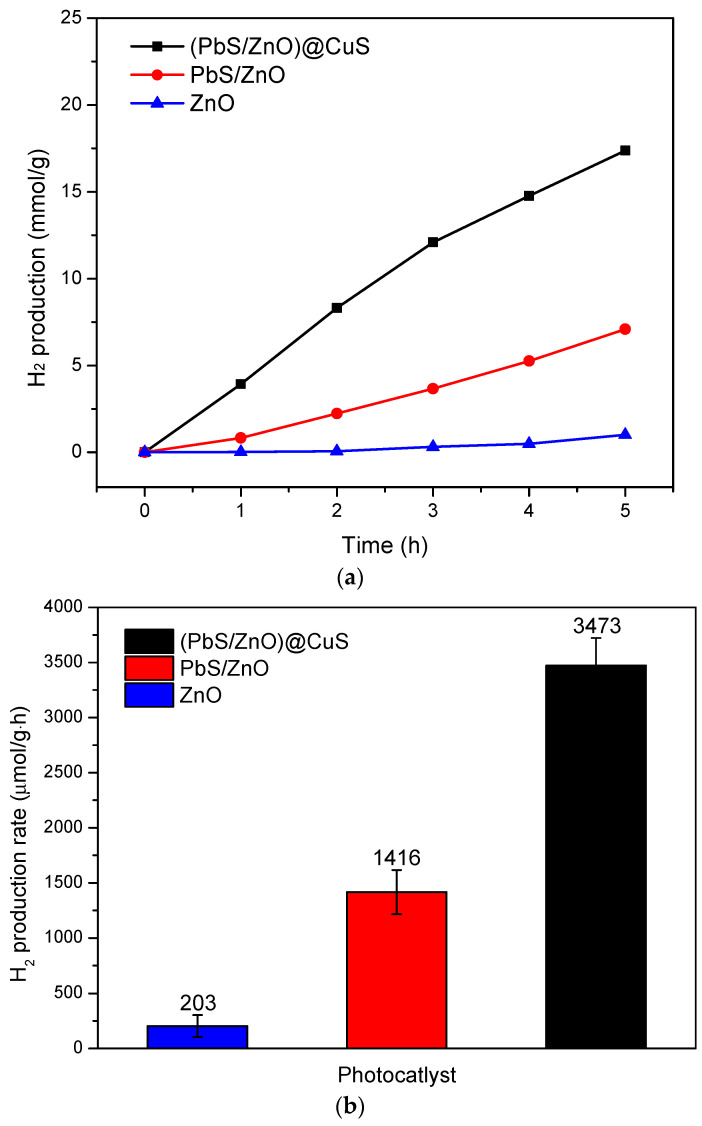
Hydrogen production efficiency of various photocatalysts in an aqueous solution containing 0.25 M Na_2_S + Na_2_SO_3_ sacrificial reagent: (**a**) relationship between hydrogen production and time; (**b**) hydrogen production rate (μmol·g^−1^·h^−1^).

The analysis of hydrogen production efficiency using photocatalysts was conducted via a solar simulator. Figure 9a displays the cumulative hydrogen production of various photocatalysts in a mixed aqueous solution containing 0.25 M Na_2_S + Na_2_SO_3_ over a duration of 5 h. It was observed that the (PbS/ZnO)@CuS photocatalyst, which underwent surface modification with sulfide PbS quantum dots and a CuS shell, along with the PbS/ZnO photocatalyst containing only QDs PbS, exhibited a significant increase in cumulative hydrogen production over time. This increase was more pronounced compared to the unmodified ZnO photocatalyst, attributed to the combination of sulfides with ZnO, which enhanced the heterogeneous photocatalyst’s energy absorption from the near-infrared to visible light regions [21,25].

As shown in Figure 9b, the hydrogen production rate of the ZnO photocatalyst without any sulfide loading was 203 μmol·g^−1^·h^−1^, while the PbS/ZnO photocatalyst had a production rate of 1416 μmol·g^−1^·h^−1^, and the (PbS/ZnO)@CuS photocatalyst exhibited a rate of 3473 μmol·g^−1^·h^−1^. In comparison, the production rate of (PbS/ZnO)@CuS was 17.1 times that of the unmodified ZnO photocatalyst. Thus, the (PbS/ZnO)@CuS photocatalyst demonstrated superior hydrogen production efficiency, owing to the ability of QDs and CuS to enhance absorption in the near-infrared to visible light regions, along with the effectiveness of the ternary (PbS/ZnO)@CuS heterostructure in reducing electron–hole pair recombination [40].

## 4. Discussion

**Figure 10:**
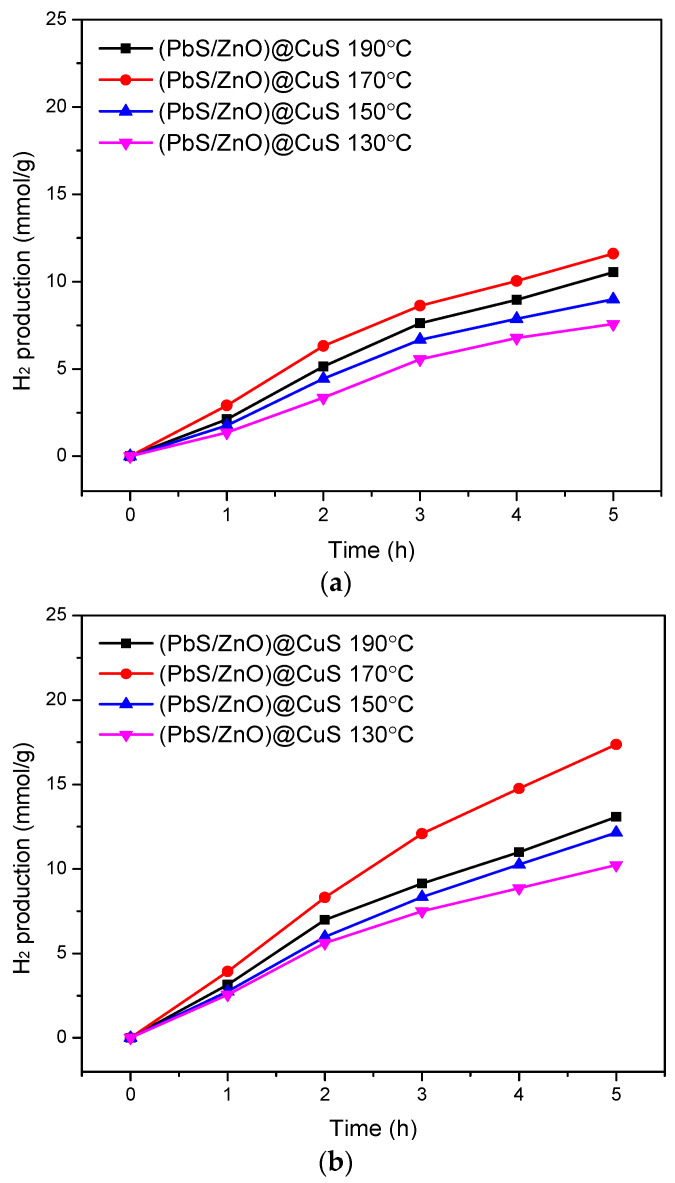
Hydrogen production efficiency of (PbS/ZnO)@CuS photocatalysts at different hydrothermal temperatures: (**a**) without the addition of Na_2_S + Na_2_SO_3_ sacrificial reagents, (**b**) with the addition of 0.25 M Na_2_S + Na_2_SO_3_ sacrificial reagents.

Figure 10 illustrates the hydrogen production efficiency analysis of the (PbS/ZnO)@CuS photocatalysts prepared at different hydrothermal temperatures, both with and without the addition of sacrificial reagents. In the absence of sacrificial reagents, the hydrogen production accumulation curves for all photocatalysts exhibited similar trends, with the photocatalyst prepared at 170 °C demonstrating the highest hydrogen production (as shown in Figure 10a). This can be attributed to the quantum confinement effect of the sulfides, which increases the energy gap and effectively reduces the occurrence of electron–hole pair recombination [23,25]. Furthermore, the photocatalyst synthesized at 170 °C had a more complete crystallinity compared to those prepared at 130 °C and 150 °C, indicating fewer lattice defects. Additionally, the 170 °C photocatalyst exhibited a higher specific surface area (as shown in Table 1), which provided more active sites for photocatalytic reactions, thereby enhancing the hydrogen production efficiency [41].

When the (PbS/ZnO)@CuS photocatalyst undergoes photocatalytic hydrogen production in a solution containing only pure water, upon exposure to light, electrons (e^−^) from the valence band of the photocatalyst are excited to the conduction band, leaving behind electron–holes (h⁺) in the valence band (Equation (1)). As the electrons migrate to the surface of the photocatalyst, they undergo a reduction reaction with hydrogen ions (H⁺) in the water, producing hydrogen gas (H₂) (Equation (2)). Meanwhile, the holes react with water molecules to produce oxygen gas (O₂) (Equation (3)).

Catalyst + hv → Catalyst (e^−^ + h^+^) (1)

2e^−^ +2H^+^ → H_2_ (2)

4h^+^ + 2H_2_O → O_2_ + 4H^+^ (3)

Wang et al. demonstrated that the addition of Na_2_S + Na_2_SO_3_ sacrificial reagents in photocatalytic aqueous solutions could effectively reduce the recombination of electron–hole pairs in the photocatalyst [42]. The use of Na_2_S generates S^2−^ ions, which are more unstable than the sulfide photocatalyst. This instability promotes the oxidation of S^2−^, thereby facilitating the oxidation of the electron–hole pairs (h^+^) while simultaneously protecting the sulfide photocatalyst from photo-corrosion. However, the oxidation of S^2−^ leads to the formation of polysulfide ions (S_n_^2−^), as represented in Equation (4), which can adversely affect hydrogen production efficiency.

The introduction of Na_2_SO_3_ results in its dissociation to produce SO₃^2−^ ions, which can convert S_n_^2−^ back to S^2−^ (Equation (5)). This conversion enables the photocatalyst to continue producing hydrogen. The efficiency of hydrogen production remains unaffected, as SO₃^2−^ also interacts with the electron–hole pairs, facilitating their removal, preventing recombination, and producing sulfate ions (SO_4_^−2^) and hydrogen ions (H^+^) (Equation (6)).

The overall reaction with the mixed sacrificial reagents can be summarized in Equation (7). The reaction mechanism when Na_2_S was mixed with Na_2_SO_3_ as sacrificial reagents is outlined as follows:

2S^2−^ + 2h^+^ → S_2_^2−^ (4)

S_2_^2−^ + SO_3_^2−^ → S_2_O_3_^2−^ + S^2−^ (5)

SO_3_^2−^ + h^+^ + 2OH^−^ → SO_4_^2−^ + 2H^+^ (6)

SO_3_^2−^ + S^2−^ + 2h^+^ → S_2_O_3_^2−^ (7)

Figure 10b illustrates the hydrogen production efficiency of various photocatalysts in a mixed aqueous solution containing 0.25 M Na_2_S and Na_2_SO_3_ as sacrificial agents. The hydrogen accumulation curves of the different photocatalysts exhibited similar trends. Specifically, the photocatalysts prepared at 130 °C, 150 °C, and 190 °C demonstrated an increase in hydrogen production efficiency from approximately 24% to 35% compared to the case without the addition of sacrificial agents. Notably, the photocatalyst synthesized at 170 °C exhibited a remarkable increase in hydrogen production efficiency of around 61%. This outcome aligns with the previously mentioned reasons, confirming that the addition of Na_2_S and Na_2_SO_3_ effectively reduces the recombination of electron–hole pairs in the photocatalyst, thereby enhancing hydrogen production efficiency.

**Figure 11:**
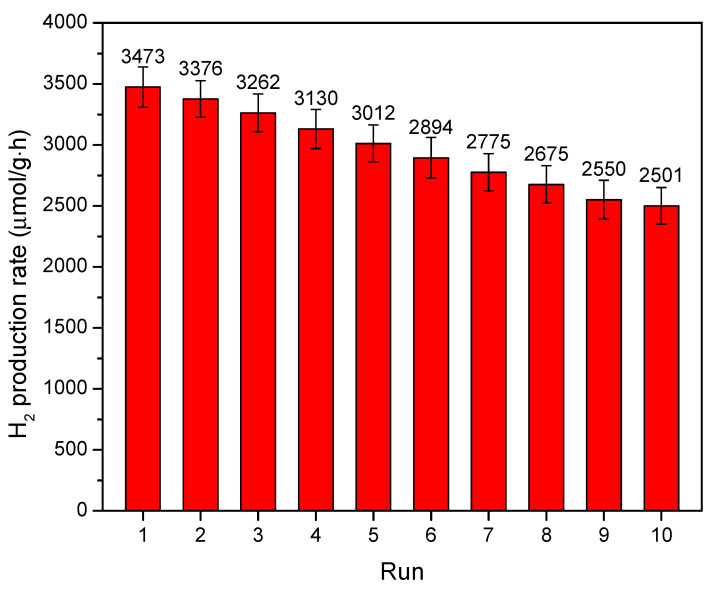
Hydrogen production of the (PbS/ZnO)@CuS photocatalyst synthesized at a hydrothermal temperature of 170 °C under 5 h of irradiation with a solar light simulator, followed by continuous cycling.

The (PbS/ZnO)@CuS photocatalyst, synthesized at a hydrothermal temperature of 170 °C, was subjected to a photocatalytic reaction under a solar light simulator for 5 h. The photocatalyst was recovered from the reaction solution through centrifugation and subsequently reintroduced into a fresh batch of mixed aqueous solution containing 0.25 M Na_2_S and Na_2_SO_3_ as sacrificial agents. This process was repeated for a total of 10 cycles to analyze hydrogen production (as shown in Figure 11). After 10 consecutive photocatalytic cycles, the (PbS/ZnO)@CuS photocatalyst maintained approximately 72% of its hydrogen production efficiency, demonstrating good stability and reusability. However, the retention rate of hydrogen production efficiency for the (PbS/ZnO)@CuS photocatalyst was slightly lower than that of the CuS/PbS/ZnO photocatalyst (with a retention rate of 78%) [25]. This discrepancy may be attributed to the higher sulfide concentration present in the core–shell-structured (PbS/ZnO)@CuS photocatalyst, leading to an increased likelihood of photo-corrosion compared to the CuS/PbS/ZnO structure [25].

## 5. Conclusions

This study successfully synthesized the core–shell-structured (PbS/ZnO)@CuS heterojunction photocatalysts by the hydrothermal method, where CuS was coated on PbS/ZnO. The results show that the quantum confinement effect of PbS quantum dots effectively enlarged the band gap, which helped reduce the recombination of electron–hole pairs. However, due to the disordered lattice arrangement of the CuS shell in the heterojunction, numerous defects were present, which may have prevented electrons and holes from effectively participating in the oxidation–reduction reactions in photocatalysis. Despite this, the (PbS/ZnO)@CuS photocatalyst synthesized at 170 °C exhibited the best hydrogen production rate. When used in a photocatalytic reaction with a mixed aqueous solution containing 0.25 M Na_2_S + Na_2_SO_3_ sacrificial agents, it achieved a hydrogen production rate of 3473 μmol·g^−1^·h^−1^. This represents a 61% increase in hydrogen production efficiency compared to the system without sacrificial agents, indicating that the heterojunction of sulfides can effectively improve the hydrogen production rate of ZnO photocatalysts.

## Figures and Tables

**Table 1:** Specific surface area, pore volume, and pore diameter of (PbS/ZnO)@CuS photocatalysts at different hydrothermal temperatures.

Photocatalyst	BET Surface Area (m^2^ g^−1^)	Pore Volume (cm^3^ g^−1^)	Pore Size (nm)
(PbS/ZnO)@CuS 130 °C	13.79	0.13	39.13
(PbS/ZnO)@CuS 150 °C	15.31	0.12	30.55
(PbS/ZnO)@CuS 170 °C	16.06	0.25	62.23
(PbS/ZnO)@CuS 190 °C	9.22	0.09	38.86

## Data Availability

The original contributions presented in this study are included in the article. Further inquiries can be directed to the corresponding author.
